# Targeting the Hippo Signaling Pathway for Tissue Regeneration and Cancer Therapy

**DOI:** 10.3390/genes7090055

**Published:** 2016-08-30

**Authors:** Wen Chun Juan, Wanjin Hong

**Affiliations:** Institute of Molecular and Cell Biology, Agency for Science, Technology and Research (A*STAR), 61 Biopolis Drive, Proteos, Singapore 138673, Singapore; wcjuan@imcb.a-star.edu.sg

**Keywords:** Hippo pathway, YAP/TAZ, TEADs, regeneration, cancer

## Abstract

The Hippo signaling pathway is a highly-conserved developmental pathway that plays an essential role in organ size control, tumor suppression, tissue regeneration and stem cell self-renewal. The YES-associated protein (YAP) and the transcriptional co-activator with PDZ-binding motif (TAZ) are two important transcriptional co-activators that are negatively regulated by the Hippo signaling pathway. By binding to transcription factors, especially the TEA domain transcription factors (TEADs), YAP and TAZ induce the expression of growth-promoting genes, which can promote organ regeneration after injury. Therefore, controlled activation of YAP and TAZ can be useful for regenerative medicine. However, aberrant activation of YAP and TAZ due to deregulation of the Hippo pathway or overexpression of YAP/TAZ and TEADs can promote cancer development. Hence, pharmacological inhibition of YAP and TAZ may be a useful approach to treat tumors with high YAP and/or TAZ activity. In this review, we present the mechanisms regulating the Hippo pathway, the role of the Hippo pathway in tissue repair and cancer, as well as a detailed analysis of the different strategies to target the Hippo signaling pathway and the genes regulated by YAP and TAZ for regenerative medicine and cancer therapy.

## 1. Introduction

The Hippo signaling pathway is an evolutionarily-conserved signaling pathway that plays an important function in organ size control, tissue regeneration, as well as tumor suppression [1]. YES-associated protein (YAP) and transcriptional co-activator with PDZ-binding motif (TAZ) are the two main downstream effectors of the Hippo signaling pathway [2]. YAP and TAZ function as transcriptional co-activators, and when they are active, they initiate a transcriptional program that enhances stem cell self-renewal and promotes cell proliferation, which are important for stimulating tissue regeneration. However, aberrant and sustained activation of YAP and TAZ can lead to the formation of malignant tumors [3]. In this review, we present an overview of the Hippo signaling pathway, its role in tissue regeneration and tumorigenesis, as well as various approaches to modulate the Hippo signaling pathway for anticancer therapy and regenerative medicine.

## 2. Overview of the Hippo Signaling Pathway

**Figure 1:**
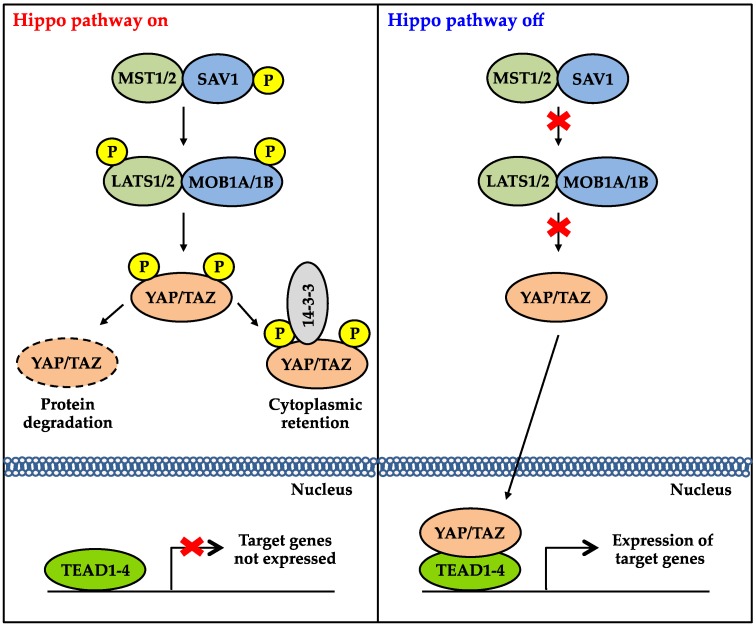
Schematic overview of the Hippo signaling pathway. (Left) When Hippo signaling is on, LATS1/2 phosphorylate YAP/TAZ, which leads to β-TrCP-mediated degradation of YAP/TAZ. Phosphorylation also promotes the retention of YAP/TAZ in the cytoplasm due to the interaction with 14-3-3. (Right) When Hippo signaling is inactivated, YAP/TAZ accumulate in the nucleus and interact with TEADs, resulting in the expression of YAP/TAZ target genes.

The core of the Hippo signaling pathway consists of a highly-conserved serine/threonine kinase cascade that negatively regulates the expression and the activity of the transcriptional co-activators, YAP and TAZ. In mammals, the core kinases involved in the pathway include mammalian STE20-like protein kinase 1 (MST1), MST2, large tumor suppressor 1 (LATS1) and LATS2 [4,5,6,7] (Figure 1). The activities of these kinases are also dependent on their interactions with scaffolding proteins. Salvador homolog 1 (SAV1) forms complexes with MST1/2, whereas MOB kinase activator 1A (MOB1A) and MOB1B interact with LATS1/2 [8,9,10]. When the Hippo pathway is activated, MST1/2 activate LATS1/2 and MOB1A/1B by phosphorylation [11]. Subsequently, LATS1/2 directly phosphorylate YAP and TAZ on HXRXXS motifs [12]. Phosphorylation inhibits YAP and TAZ activities by two main mechanisms (Figure 1). First, phosphorylation of YAP on serine 127 and TAZ on serine 89 create binding sites for 14-3-3, in which interactions with 14-3-3 promote cytoplasmic retention of YAP and TAZ [13,14]. Second, phosphorylation of YAP on serine 381 and TAZ on serine 311 promotes another phosphorylation event mediated by casein kinase 1. This additional phosphorylation event activates a phosphodegron that is targeted by β-transducin repeat-containing protein (β-TrCP), leading to the degradation of YAP and TAZ proteins [12,15]. As a result, YAP and TAZ accumulate in the nucleus and promote gene expression when the Hippo pathway is not active (Figure 1). YAP and TAZ do not contain a DNA-binding domain. Therefore, they have to form complexes with other transcription factors to modulate gene expression. In mammals, TEA domain transcription factors (TEAD1 to TEAD4) are the major transcription factors that bind to YAP and TAZ [16,17,18,19]. Other transcription factors, such as T-box transcription factor 5 (TBX5) [20,21], p73 [22], RUNT-related transcription factor 1 (RUNX1) and RUNX2 [23], have also been shown to interact with YAP and TAZ to modulate gene expression.

## 3. Regulation of the Hippo Signaling Pathway

Currently, several upstream regulators of the Hippo signaling pathway have been identified, which include: (1) proteins that determine cell polarity; (2) adherens and tight junctions; (3) cross-talk between other signaling pathways; and (4) mechanical cues. Summaries of these upstream regulators are outlined below.

### 3.1. Regulation by Proteins Involved in Cell Polarity

**Figure 2:**
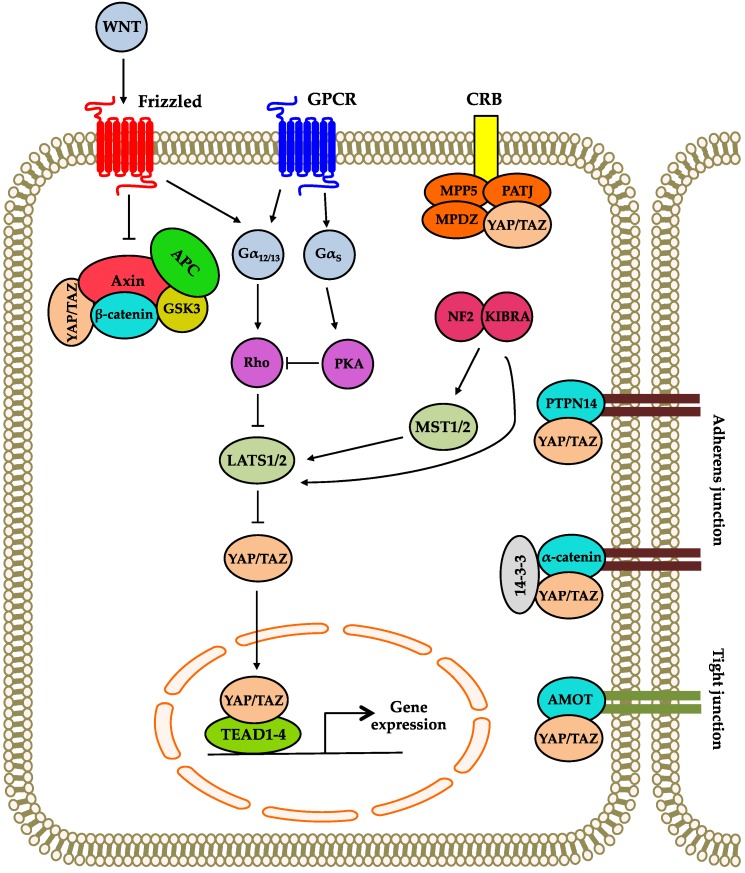
Regulation of the Hippo signaling pathway in mammalian cells. The Hippo pathway is regulated by several upstream regulators: (1) proteins involved in cell polarity; (2) WNT and GPCR signaling; (3) components of the adherens and tight junctions. Pointed arrowheads represent activating interactions, whereas blunt arrowheads represent interactions that are inhibitory.

Cell polarity refers to the asymmetric organization of cellular organelles, structures and proteins, which allow eukaryotic cells to perform highly-specialized functions [24]. Crumbs (Crb) is a transmembrane protein that is essential for establishing the apical-basal polarity of a cell. In *Drosophila*, Crb has been identified to be an important upstream regulator of the Hippo signaling pathway. Mechanistically, Crb directly interacts with the apical membrane-associated protein, Expanded (Ex), which may result in the recruitment and the activation of the core Hippo kinases [25,26,27]. Functionally, the interactions between Crb, Ex and the core Hippo kinases at the cell membrane play an important role in the collective migration of *Drosophila* border cells. However, phosphorylation of Yorkie, the *Drosophila* homolog of YAP, by Warts does not promote border cell migration. Instead, it provides negative feedback to control the speed of migration [28]. In mammals, YAP and TAZ also interact with members of the Crumbs complex, such as membrane palmitoylated protein 5 (MPP5), PATJ, AMOT and multiple PDZ domain Crumbs cell polarity complex component (MPDZ) (Figure 2). Interaction with the Crumbs complex promotes the phosphorylation and the accumulation of YAP and TAZ in the cytoplasm [29].

Neurofibromin 2 (NF2) and kidney and brain protein (KIBRA) are important regulators of Hippo signaling, which localize to the apical domain of polarized epithelial cells [30,31]. Earlier studies in *Drosophila* and mammalian cells have demonstrated that NF2 and KIBRA can promote Hippo signaling by acting directly on MST1/2 and LATS1/2 [32,33,34] (Figure 2).

### 3.2. Regulation by Adherens and Tight Junctions

Proteins found in adherens and tight junctions are important upstream regulators of the Hippo signaling pathway (Figure 2). A recent study has demonstrated that E-cadherin inhibits the localization of YAP in the nucleus in a process that is dependent on the components of the Hippo signaling pathway, such as LATS1/2 [35]. α-catenin, a component of the adherens junction, is also found to be a negative regulator of YAP [36,37]. In keratinocytes and the hair follicle stem cell compartment, YAP forms a complex with 14-3-3 and α-catenin. This complex suppresses the activity of YAP by inhibiting the localization of YAP in the nucleus (Figure 2) and the dephosphorylation of YAP at serine 127 by protein phosphatase 2A (PP2A) [36,37]. Protein tyrosine phosphatase type 14 (PTPN14) has also been shown to inhibit YAP transcriptional activities. Mechanistically, PTPN14 interacts and localizes YAP in the cytoplasm (Figure 2), whereby the interaction between the two proteins is mediated by the PPXY motifs of PTPN14 and the WW domains of YAP [38,39,40].

The angiomotin (AMOT) family of proteins consists of three members, AMOT, AMOTL1 and AMOTL2, which localize to tight junctions, as well as the actin cytoskeleton [41]. Functionally, these proteins have been shown to regulate cell proliferation and cell migration [42,43]. Several studies have demonstrated that the AMOT family of proteins can directly interact with and suppress the transcriptional activities of YAP and TAZ by two different mechanisms. One mechanism that has been proposed is that AMOT inhibits YAP and TAZ via a LATS-independent manner by sequestering them at the tight junctions and the actin cytoskeleton [44,45,46] (Figure 2). Another mechanism that has been suggested is that AMOT promotes the inhibitory phosphorylation of YAP and TAZ by functioning as a scaffold to recruit LATS [45].

### 3.3. Regulation by Cross-Talk between Other Signaling Pathways

Previous studies have shown that extracellular ligands interact with G-protein-coupled receptors (GPCRs) to regulate the Hippo signaling pathway. Sphingosine-1-phosphate (S1P), thrombin, lysophosphatidic acid (LPA) and estrogen have been shown to activate YAP and TAZ via GPCRs coupled to Gα_12/13_ or Gα_q/11_ [47,48,49]. Mechanistically, Gα_12/13_ act through Rho GTPases and F-actin to inhibit LATS1/2 via a mechanism that does not depend on MST1/2. In contrast, stimulating GPCRs coupled to Gα_s_ by soluble factors such as adrenaline and glucagon can inhibit YAP activity by signaling through protein kinase A (PKA) [47,48,49,50] (Figure 2).

Wingless-type MMTV integration site (WNT) signaling has also been shown to regulate YAP and TAZ activity, as well (Figure 2). Azzolin et al. (2014) have demonstrated that in the absence of WNT stimulation, YAP and TAZ are sequestered in the cytoplasm by the β-catenin destruction complex, which consists of adenomatous polyposis coli (APC), axin and glycogen synthase kinase 3 (GSK3) [51]. Importantly, stimulation with WNTs inactivates the β-catenin destruction complex, which leads to the accumulation of YAP/TAZ in the nucleus and the expression of their target genes. Interestingly, Park et al. (2015) found that WNTs enhance the activation of YAP and TAZ through an alternative WNT signaling pathway that signals through Gα_12/13_, Rho GTPases and LATS, but not through the β-catenin destruction complex [52]. The findings by Park et al. (2015) are consistent with an earlier report that showed that mutations in APC can activate YAP through a mechanism that does not involve the β-catenin destruction complex [53]. Taken together, these results indicate that WNT signaling can utilize multiple mechanisms to activate YAP and TAZ and that the actual mechanism(s) utilized is highly dependent on the cellular context. There are also other signaling pathways that interact with the Hippo signaling pathway. These include phosphatidylinositol-4,5-bisphosphate 3-kinase (PI3K) [54], transforming growth factor-β (TGFβ) [55] and Notch signaling [56].

The Hippo signaling pathway is also regulated by the metabolic status of the cell. Under conditions of cellular energy stress, such as glucose deprivation, the AMP-activated protein kinase (AMPK) becomes active and directly phosphorylates YAP at serine 94 [57,58]. This leads to the suppression of YAP activity because phosphorylation of YAP at serine 94 disrupts the interaction between YAP and TEADs. Furthermore, energy deprivation also increases the activity of LATS1/2, which can lead to inhibition of YAP activity, as well [57,58].

The expressions of YAP and TAZ are also regulated by oxygen concentration. Under hypoxia, hypoxia-inducible factor 1-alpha (HIF1α) can directly promote the transcription of TAZ [59]. In addition, the expression of the E3 ubiquitin ligase, SIAH2, is enhanced by HIF1α. SIAH2 promotes the degradation of LATS2, which leads to the activation of YAP and TAZ [59,60]. Because hypoxia may suppress tumor growth, these observations suggest that the activation of YAP and TAZ under hypoxia could function as a means for cancer cells to grow under hypoxic conditions.

### 3.4. Regulation by Mechanical Cues

Cells in vivo are frequently exposed to physical and mechanical signals from the tissue microenvironment. The cells respond to these mechanical cues by remodeling the actin cytoskeleton, as well as activating certain gene transcription programs [61,62]. As a result, the responses to these mechanical cues can regulate different types of cell behavior, such as cell growth, cell differentiation and programmed cell death (apoptosis). Cell density is one of the first physical signals discovered, that can regulate YAP and TAZ activity [13]. When cells are seeded at a low cell density, YAP and TAZ are observed to accumulate in the nucleus and activate transcription of their target genes. In contrast, YAP and TAZ localize in the cytoplasm instead when cells are grown at a high cell density [13]. Subsequently, several reports have also shown that YAP and TAZ can be regulated by other mechanical signals, such as the stiffness of the extracellular matrix and cell geometry. When cells are grown on a stiff matrix, YAP and TAZ become active and accumulate in the nucleus, which drive the expression of their target genes, such as connective tissue growth factor (*CTGF*) and ankyrin repeat domain 1 (*ANKRD1*). Conversely, when cells are grown on a soft matrix, YAP and TAZ become inactive and localize to the cytoplasm instead [63]. Cell geometry also regulates the activity of YAP and TAZ, as well. Dupont et al. (2011) and Wada et al. (2011) demonstrated that YAP and TAZ are predominantly localized to the nucleus in cells that experience a high degree of cell spreading. In contrast, YAP and TAZ are observed to be in the cytoplasm in cells that are more compact [63,64]. Mechanistically, the localization of YAP and TAZ in response to mechanical cues is dependent on the tension of the actin cytoskeleton and the activity of Rho GTPases [63,65].

Apart from mechanical cues, the other upstream regulators of Hippo signaling, which include adherens and tight junctions, cell polarity and GPCR signaling, also converge to regulate Hippo signaling via the actin cytoskeleton. For example, Gjorevski et al. (2012) observed that actin bundles are connected to apical junctions by interacting with other adaptor proteins, such as catenins, AMOT and NF2 [66]. Furthermore, these adaptor proteins also modulate the activity of actin dynamics [67]. Therefore, the regulation of YAP/TAZ activity by AMOT, catenins and NF2 could be mediated by actin rearrangements. Ligands for GPCRs coupled to Gα_12/13_, such as LPA and S1P, have been observed to generate contractive actin bundles. Conversely, the induction of actin bundles can be reversed by ligands for GPCRs coupled to Gα_s_ [49,68]. Taken together, these observations suggest that the actin cytoskeleton plays an important role to integrate the upstream stimuli to the Hippo signaling pathway.

## 4. The Role of YAP and TAZ in Cancer

Growth regulation is one of the most important functions of the Hippo signaling pathway in normal cell physiology [69,70]. In *Drosophila*, mutations of the Hippo pathway core kinases or ectopic expression of Yorkie lead to overgrowth of organs, such as the wings and the eyes. Similar observations were also made in mice. For example, ectopic expression of *Yap* specifically in the liver can lead to an enlarged liver. Notably, the liver reverts back to its normal size when *Yap* overexpression is switched off [71,72]. Similarly, liver enlargement is also observed in mice with knockout of *Mst1/2* [73]. However, there is no discernable change in the size of the kidney, intestines and lung in *Mst1/2* knockout mice. These observations suggest that the role of the Hippo signaling pathway in organ size control is conserved in some, but not in all mammalian tissues.

Aberrant Hippo signaling leading to abnormal activation of YAP and TAZ is frequently observed in human cancers [3,74,75,76]. These observations suggest that hyperactivation of YAP and TAZ could play a role in the development and the progression of cancer. Definitive evidence for the role of YAP and TAZ in tumorigenesis and disease progression has been shown in cell culture and animal models. Here, overexpression of YAP or TAZ confers a proliferative advantage, promotes cell invasion and migration and enhances cancer stem cell characteristics in vitro [13,77,78,79,80]. Importantly, activation of YAP by abrogating Hippo signaling, such as knocking out *Mst1* and *Mst2* in the mouse liver, has been shown to be sufficient to drive tumor formation in mice [73,81,82]. Conversely, loss of YAP or TAZ in cancer cell lines inhibits anchorage-independent growth, reduces cell migration and invasion and blocks the self-renewal capability of cancer stem cells.

There is strong evidence to show that the transcriptional activity of YAP and TAZ is dependent on their interactions with TEADs to confer cancer cell phenotypes. To start, YAP and TAZ could not induce oncogenic transformation if mutations are generated, which prevent them from interacting with TEADs [17,83,84,85]. Furthermore, the fusion of TEAD to a YAP or TAZ mutant that could not interact with endogenous TEADs can restore transforming activity [17,83,84]. Recent ChIP-seq analyses have begun to shed some light regarding the transcriptional program of YAP and TAZ. These groups have observed that a large proportion of YAP/TAZ/TEAD target genes are associated with biological processes related to cell cycle progression, regulation of cell migration and extracellular matrix organization [86,87]. Apart from TEADs, YAP and TAZ also interact with other transcription factors, such as SMADs and TBX5, to promote tumorigenesis [20,21,88].

Downregulation of global microRNA (miRNA) expression is a common feature in human cancers, which may play a role in tumorigenesis [89,90,91,92]. Recent studies suggest that YAP inhibits the biogenesis of mature miRNAs by a mechanism that is dependent on cell density [92,93]. When cells are seeded at a low density, YAP accumulates in the nucleus and sequesters DEAD box helicase 17 (DDX17), an important regulator of miRNA biogenesis, resulting in a decreased expression of mature miRNAs [93]. At a high cell density, YAP is retained in the cytoplasm, which promotes the association of DDX17 with other factors of the miRNA-processing machinery. Interestingly, a constitutively-active mutant of YAP that could not interact with TEADs (YAP S94A/5SA) could still repress miRNA biogenesis and promote cell growth. These results suggest that YAP suppresses the production of mature miRNAs in a TEAD-independent manner and that YAP also possesses a TEAD-independent mechanism to enhance cell proliferation. Indeed, the oncogene MYC was observed to be upregulated in a posttranscriptional manner as a result of YAP-mediated downregulation of miRNAs that regulate MYC expression [93].

Human tumors are made up of multiple distinct cell subpopulations that have different growth and metastatic properties [94,95]. Cancer stem cells are defined as a subpopulation of tumor cells within the original tumor that are capable of self-renewal and have the ability to give rise to another tumor, with some of the heterogeneity of the original tumor, when these cells are transplanted into immunocompromised mice [96]. YAP/TAZ has been shown to promote cancer stem cell characteristics [79,97,98,99]. For instance, forced expression of constitutively-active TAZ in non-malignant mammary epithelial cells is sufficient to confer cancer stem cell characteristics. In contrast, knockdown of TAZ inhibits the self-renewal and tumor-initiating potential of breast cancer cells. Significantly, TAZ is highly expressed in high-grade human primary breast cancer samples that are poorly differentiated and also express embryonic and normal mammary stem cell genes [79]. Nuclear TAZ is also highly expressed in high-grade glioblastomas [80]. Forced expression of TAZ enhances cell invasion, self-renewal and the tumor-initiating property similar to mesenchymal-like stem cells. In contrast, downregulation of TAZ in glioma stem cells suppresses invasion, self-renewal and the tumor-initiating property [80]. Taken together, these observations strongly argue for a crucial role of TAZ in breast cancer and glioma stem cells. YAP has been shown to be overexpressed in esophageal cancer cells [99]. Ectopic expression of YAP confers cancer stem cell properties, such as sphere- and tumor-initiating capacity, in non-transformed murine esophageal epithelial cells. Mechanistically, YAP confers cancer stem properties in esophageal cancer cells by direct upregulation of SOX9 [99].

Interestingly, there is evidence to suggest that YAP can function as a tumor suppressor depending on the cell and tissue context. For instance, the activation of DNA damage-induced apoptosis in hematological cancers, such as multiple myeloma and leukemia, is dependent on the interaction between nuclear ABL1 kinase and YAP [100]. Furthermore, low expression of YAP is frequently observed in hematologic malignancies. Importantly, shRNA knockdown of MST1 enhances YAP expression and triggers apoptosis in multiple myeloma cells [100].

## 5. Mechanisms of Activating YAP and TAZ in Cancer

It is critical to elucidate the mechanisms of activating YAP and TAZ in cancer because this could lead to the development of novel therapeutics to treat the disease. Since the Hippo signaling pathway suppresses the oncogenic activities of YAP and TAZ, it has been hypothesized that the core components of the Hippo pathway are frequently mutated in cancer. Strikingly, very few inactivating mutations of the Hippo pathway components have been identified, except for *LATS2* and *SAV1* in malignant mesothelioma [101], as well as mutations in *NF2* in neurofibromatosis [102,103]. Instead of harboring inactivating mutations of the Hippo pathway components, YAP and TAZ are frequently overexpressed in human cancers. Gene amplification is a mechanism for overexpressing YAP and TAZ in oral squamous cell carcinoma and ependymomas [104,105,106]. Interestingly, gene amplification of YAP and TAZ is not commonly observed in liver and breast cancers, even though the protein levels of YAP and TAZ are elevated [78,79,107]. These observations suggest that in some cancers, YAP and TAZ overexpression is likely to occur at the transcriptional and posttranscriptional levels.

Activation of YAP and TAZ can also occur due to mutations in other signaling pathways, such as the GPCR signaling pathway. Two recent studies have shown that activating mutations in the genes that encode for Gα_q_ and Gα_11_ are observed in approximately 80% of uveal melanomas [108,109]. These mutations drive the development of uveal melanoma by activating YAP via LATS-dependent and LATS-independent mechanisms. Importantly, the use of a YAP inhibitor can suppress the growth of uveal melanoma cells both in vitro and in vivo, which strongly supports the notion of targeting YAP to treat uveal melanomas with Gα_q_ and Gα_11_ mutations [108,109].

Earlier studies have shown that the oncogene, Kirsten rat sarcoma viral oncogene homolog (*KRAS*), is commonly mutated in human malignancies. However, targeting KRAS remains a challenge, and hence, significant efforts are being made to target effectors downstream of KRAS [110]. One group has observed that constitutively-active KRAS promotes the post-translational modification of YAP and enhances its transcriptional activity via the mitogen-activated protein kinase (MAPK) pathway [111]. Activation of YAP is crucial in KRAS-driven colon and lung cancer because KRAS and YAP converge to regulate epithelial-mesenchymal transition [112]. Intriguingly, coexpression of oncogenic KRAS with a YAP mutant that is refractory to LATS phosphorylation could further stimulate the expression of YAP target genes, such as *CTGF* and cysteine-rich angiogenic inducer 61 (*CYR61*). These observations suggest that oncogenic KRAS can enhance the transcriptional activity of YAP through a mechanism that is independent of the Hippo signaling pathway. Crucially, loss of YAP in the pancreas inhibits the development of pancreatic ductal adenocarcinoma in a genetically-engineered Kras mouse model [111], implying that YAP is a promising target for cancers harboring activating mutations in *KRAS*. Finally, YAP can also be overexpressed as a form of escape mechanism from oncogenic KRAS addiction in pancreatic cancer. Mechanistically, YAP cooperates with E2F transcription factors to activate a cell cycle and DNA replication program to promote tumor maintenance that does not depend on KRAS expression [113].

## 6. Hippo Signaling in Organ Regeneration

Embryonic stem cells (ESCs) are stem cells obtained from the inner cell mass of a human embryo. These cells are pluripotent, which means that ESCs have the capability to differentiate into all of the different cell types of the human body. Therefore, ESCs have a tremendous potential to be used for therapeutic purposes in regenerative medicine [114]. Transcriptional profiling of embryonic and adult stem cells revealed that *Yap* and *Tead2* are highly expressed in ESCs, hematopoietic stem cells, as well as neural stem cells [115]. In contrast, *Yap* expression is downregulated when ESCs are undergoing differentiation [116]. These observations suggest that Yap and Teads could play a role in maintaining pluripotency. Indeed, genome-wide analysis of Yap target genes in murine ESCs has shown that Yap directly regulates genes that promote pluripotency, such as SRY-box 2 (*Sox2*) and POU class 5 homeobox 1 (*Oct4*) [116]. Furthermore, YAP expression also increases during the reprogramming of normal human fibroblasts into induced pluripotent stem cells [116].

In addition, several studies have shown that YAP and TAZ can crosstalk with other signaling pathways to maintain stemness. Tamm et al. (2011) have observed that Yap is activated downstream of leukemia inhibitory factor (Lif) signaling in murine ESCs. This process enhances Yap-dependent transcription of pluripotency genes, such as *Oct3/4* and *Nanog* [117]. The TGFβ signaling pathway is another important regulator of stemness in ESCs. SMAD proteins are downstream effectors of the TGFβ pathway by functioning as transcription factors [118]. Varelas et al. (2008) have demonstrated that TAZ interacts with SMAD2/3 and promotes the localization of SMAD2/3 in the nucleus, which enhances the expression of genes that regulate pluripotency [55]. 

Organ regeneration is a highly complex process, which involves differentiation of stem cells followed by active cell proliferation of the differentiated cells to replace the tissue that is lost during injury [119]. However, the activation of stem cells is not required for regeneration in all tissues. For instance, the heart and the liver regenerate from differentiated cells instead of stem cells [120,121]. There is an increasing amount of evidence to suggest that the Hippo signaling pathway, especially Yap, plays a critical role in regulating organ regeneration across different species. Forced expression of a dominant-negative mutant of Yap in *Xenopus* tadpoles impedes the regeneration of the hindlimb after amputation [122]. In *M. lignano*, a certain species of flatworm, knockdown of the core components of the Hippo signaling pathway promotes regeneration after cutting. Conversely, the flatworms fail to regenerate after knockdown of Yap by RNA interference (RNAi) [123].

The importance of Hippo signaling in regeneration has also been observed in mammals. Heallen et al. (2013) have shown that conditional knockout of *Salvador* to inactivate Hippo signaling promotes heart regeneration in mice after resection of the cardiac apex, as well as after myocardial infarction [124]. Importantly, transgenic mice that express an active form of Yap can regenerate more effectively than wildtype mice after myocardial infarction [125]. Mechanistically, activated Yap enhances heart regeneration in mice by promoting the proliferation of cardiomyocytes [125].

The mammalian liver is an organ that has a huge capacity to regenerate. Using a rat partial hepatectomy model, a study has observed that Mst1/2 and Lats1/2 are inhibited during liver regeneration, which are associated with an increase in total Yap protein expression [126]. Subsequently, the activities of the Hippo core kinases return to normal when the liver returns to its normal size [126]. Expression of Yap is also upregulated at the mRNA level during liver regeneration by the transcription factor, GA-binding protein (Gabp) [127]. Gabp is a direct regulator of *Yap* mRNA expression in mice by binding to the *Yap* promoter. Forced expression of Gabp enhances the proliferation of hepatocytes. Furthermore, expression of Gabp and Yap increases after partial hepatectomy. Collectively, these results imply that Gabp-mediated upregulation of Yap is important for liver regeneration [127].

The role of Hippo signaling is also studied in intestinal regeneration [128]. Using whole-body irradiation to evaluate intestinal regeneration in mice, Gregorieff et al. (2015) have observed that YAP becomes active in intestinal epithelial cells less than five days post-irradiation [129]. A decrease in crypt proliferation after irradiation is also observed in mice with knockout of *Yap* specifically in intestinal epithelial cells and intestinal stem cells. Mechanistically, Gregorieff et al. (2015) also found that YAP promotes early intestinal regeneration by upregulating the EGFR ligand, epiregulin [129]. Intriguingly, loss of YAP in intestinal epithelial cells promotes tissue hyperplasia in the long term by hyperactivating WNT signaling [130]. Colitis caused by dextran sulfate sodium (DSS) is another approach to study intestinal regeneration. Mice with knockout of *Yap* in intestinal epithelial cells demonstrated a higher mortality rate and a more extensive loss of crypt compartments when compared to wildtype mice after DSS-induced colitis and regeneration [131,132]. Mechanistically, interleukin-6 receptor subunit beta activates YAP to promote intestinal regeneration after DSS-induced injury [132]. Taken together, these observations suggest that YAP and WNT signaling work together to promote intestinal regeneration after injury, whereby YAP plays a more important role during the first few days of regeneration.

## 7. Pharmacologic Manipulation of the Hippo Signaling Pathway

The studies described above suggest that controlled activation of YAP and TAZ could be therapeutically useful in regenerative medicine. In contrast, blocking the activity of YAP and TAZ may benefit patients with cancer. Below we will describe some of the therapeutic approaches to modulate the activity of YAP and TAZ and their target genes (Table 1).

### 7.1. Disrupting YAP-TEAD and TAZ-TEAD Interactions

The downstream effectors of the Hippo signaling pathway, YAP and TAZ, are frequently overexpressed in numerous cancers [75,77,78,155,156,157]. Functionally, animal models have demonstrated that elevated expression of YAP or TAZ is sufficient to promote tumor formation in the breast, liver and colon [71,77,78,81,82,158]. Furthermore, many of the oncogenic activities of YAP and TAZ are dependent on their association with the TEAD proteins [84,133,159]. Therefore, the development of pharmacologic compounds to disrupt the YAP-TEAD and the TAZ-TEAD complexes is a rational approach to treat human cancers with hyperactive YAP or TAZ activity.

A recent high throughput screening has identified three molecules from the porphyrin family as top hits for inhibiting the Gal4-TEAD4 luciferase reporter assay. Among these three compounds, verteporfin is found to be the most effective in disrupting the interaction between YAP and TEADs, as well as inhibiting liver overgrowth due to YAP overexpression [133]. However, verteporfin is also toxic to non-malignant cells and has low aqueous solubility, which may limit its use in vivo. Flufenamic acid, a non-steroidal anti-inflammatory drug, is identified in another drug screen that can also interfere with the interaction between YAP and TEADs [134]. Mechanistically, X-ray crystallography analyses have shown that flufenamic acid binds to the hydrophobic central pocket of TEADs, which is crucial for the interaction with YAP. More importantly, functional studies demonstrated that flufenamic acid could suppress YAP-dependent transcription, cell migration and proliferation [134]. Taken together, these studies suggest that the development of small molecules to disrupt the interactions between YAP/TAZ and TEADs may be a useful approach to treat YAP- and TAZ-driven cancers.

The use of peptide-based compounds is another strategy to inhibit YAP-TEAD and TAZ-TEAD interactions. X-ray crystallography studies have shown that YAP residues 86–100 bind to the hydrophobic pocket on the surface of TEADs [135]. Furthermore, these residues are the most critical for mediating interactions between YAP and TEADs [135]. Therefore, designing and optimizing a peptide based on YAP residues 86–100 could potentially inhibit YAP-TEAD interactions. By performing a truncation analysis and an alanine scan of YAP residues 81–100, Zhang et al. (2014) have designed a potent cyclic peptide inhibitor of YAP-TEAD interactions [136]. It will be interesting to test whether this cyclic peptide can inhibit YAP-dependent tumor growth.

Vestigial-like family member 4 (VGLL4) is a transcriptional regulator that functions by interacting with TEADs via its Tondu domains [160,161]. By directly competing with YAP and TAZ for binding TEADs, VGLL4 has been shown to repress YAP-dependent tumor growth [137]. Based on the interactions between VGLL4 and TEAD4, a peptide has been developed to disrupt YAP-TEAD complexes. Crucially, treating primary gastric cancer cells with this peptide can inhibit tumor growth, demonstrating the therapeutic potential of using peptide-based inhibitors to treat human cancers with hyperactive YAP or TAZ activity [137]. However, it is important to note that the cost of manufacturing peptide-based compounds is high. Furthermore, peptide-based compounds are degraded quickly by enzymes, which make them difficult to administer in vivo.

### 7.2. Tankyrase Inhibitors

The AMOT family of proteins has been shown to negatively regulate the oncogenic properties of YAP and TAZ by inhibiting nuclear localization through direct protein-protein interactions and by enhancing the phosphorylation of YAP and TAZ by activating LATS1/2 [44,45,46,162]. These findings suggest that regulating the expression levels of AMOT could be another avenue to inhibit YAP and TAZ in cancer. A recent report found that tankyrase inhibitors could target YAP indirectly. Mechanistically, tankyrases interact with AMOT and enhance their degradation through the E3 ligase RNF146 [138]. As a result, tankyrase inhibitors antagonize YAP activity by stabilizing AMOTs. There are several tankyrase inhibitors available, such as XAV939, inhibitor of WNT response 1 (IWR-1), G007-LK and G244-LM [139,140]. It will be of great interest to assess whether these tankyrase inhibitors are capable of treating tumors with high YAP or TAZ activity.

### 7.3. Inhibiting YAP and TAZ Target Genes

Earlier studies have shown that YAP and TAZ promote tumorigenesis and metastasis by regulating the expression of genes that are dependent on TEADs [17,84,159]. Therefore, a possible approach to treat YAP/TAZ-dependent tumors is to develop strategies to inhibit the target genes of YAP and TAZ.

CYR61 and CTGF belong to a family of secreted cysteine-rich proteins that regulate a plethora of biological processes, including cell migration, cell proliferation and cell adhesion [163,164]. Because CYR61 and CTGF play a positive role in cell proliferation, therefore, it is not surprising that these two proteins are overexpressed in numerous cancers, including pancreatic cancer [165], gliomas [166], prostate cancer [167] and breast cancer [168]. Several studies have shown that CYR61 and CTGF are direct targets of YAP and TAZ [17,86,87,169]. These observations suggest that antagonizing the activity of CYR61 and CTGF could be a potential approach to treat YAP- and TAZ-dependent tumors.

FG-3019 and 093G9 are monoclonal antibodies that target CTGF and CYR61, respectively. The use of these antibodies as a single agent led to a reduction in tumor growth in mouse models of pancreatic and breast cancer [141,142]. Since siRNA knockdown of CTGF or CYR61 has been shown to inhibit the growth of transformed mammary epithelial cells that express high levels of YAP or TAZ, it will be interesting to test whether these antibodies have an effect on YAP/TAZ-dependent tumors in humans.

The oncogenic receptor tyrosine kinase, AXL, is also identified as an important downstream target of YAP. RNAi-mediated knockdown of AXL inhibited cell survival and cell invasion in liver cancer cell lines that express high levels of YAP [170]. There are currently several tyrosine kinase inhibitors that target AXL, such as foretinib and sunitinib [143]. Additional clinical studies are required to assess whether these AXL inhibitors have a therapeutic effect on YAP/TAZ-dependent cancers.

Resistance to tyrosine kinase inhibitors is a major hurdle for the treatment of kinase-driven cancers [171]. Through a shRNA screen, YAP was shown to be a critical mediator of resistance towards RAF and mitogen-activated protein kinase kinase (MEK) inhibitors in a lung cancer cell line that harbor activating mutations in *BRAF* [144]. Mechanistically, YAP promotes resistance to RAF and MEK inhibitors by transcriptionally upregulating the antiapoptotic protein, BCL2 like 1 (BCL-XL). Importantly, resistance to RAF and MEK inhibitors mediated by YAP can be overcome by using the BCL-XL inhibitor, navitoclax (ABT-263) [144].

Interactions between the tumor and the tumor microenvironment have increasingly been recognized to play a critical role in tumor progression and resistance to anticancer agents [172]. Infiltrating cells of the immune system are an important component of the tumor microenvironment [173]. These tumor-associated leukocytes drive tumor growth by releasing growth factors, such as epidermal growth factor (EGF), vascular endothelial growth factor (VEGF) and fibroblast growth factor 2 (FGF2). Furthermore, these immune cells also drive metastasis by releasing enzymes that degrade the extracellular matrix, such as matrix metallopeptidase 9 (MMP-9) [172,174,175]. Therefore, understanding how tumors recruit infiltrating immune cells is crucial for the development of new therapies to treat both primary and secondary tumors. A recent study demonstrated that hyperactivated YAP signaling drives the recruitment of myeloid-derived suppressor cells, which promotes tumor progression in a mouse model of prostate cancer. Mechanistically, YAP upregulates the expression of C-X-C motif chemokine 5 (CXCL5) in tumor cells to recruit myeloid-derived suppressor cells, and appropriately, the use of antibodies to target CXCL5 suppresses tumor progression [176]. Taken together, these results suggest that the inhibition of CXCL5 signaling may have a therapeutic value in prostate cancers with elevated YAP or TAZ activity.

Amphiregulin (AREG) is a glycoprotein that can bind and activate the epidermal growth factor receptor (EGFR). AREG is initially translated as a 252-amino acid protein, which needs to undergo proteolytic cleavage at the cell membrane before it is shed into the microenvironment [177]. Functional studies using cancer cell lines have shown that AREG is involved in several hallmarks of cancer, such as inhibiting apoptosis [178], promoting cancer cell invasion and metastasis [179], enhancing angiogenesis [180] and endowing cancer cells with unlimited replicative potential [181]. Clinically, AREG is overexpressed in different types of cancers, such as breast, lung and colon cancers [182,183,184]. A study has shown that AREG is a direct transcriptional target of YAP. Interestingly, YAP-induced secretion of AREG enhances the proliferation of neighboring cells in a non-cell-autonomous manner [185]. These results indicate that AREG may be a promising target for treating cancers with increased YAP or TAZ activity. The development of neutralizing antibodies against AREG is one approach to target AREG. Remarkably, these anti-AREG antibodies have been shown to suppress the growth of ovarian cancer cells in a xenograft model [186]. Another approach to target AREG is to inhibit the proteases that are responsible for the cleaving and the shedding of AREG. A disintegrin and metalloprotease 17 (ADAM17) is a metalloprotease that is responsible for the proteolytic cleavage and the shedding of AREG [187]. A highly selective antibody against ADAM17 has been developed, which can inhibit the proteolytic cleavage of AREG both in vitro and in vivo, as well as decreasing the growth of ovarian tumors in mice [188,189]. It will be of great interest to assess whether these antibodies against AREG and ADAM17 are effective in tumors that have elevated YAP or TAZ activity.

### 7.4. Targeting the WW Domains of YAP and TAZ

Analyses of the protein sequences of YAP and TAZ have revealed that YAP contains two WW domains, whereas TAZ contains one WW domain only [190,191]. The WW domain mediates protein-protein interactions by binding to PPXY motifs [192,193]. Functionally, the WW domains are essential for the oncogenic properties of YAP and TAZ because mutating the WW domains suppresses the ability of YAP and TAZ to promote oncogenic transformation and transcription. Further studies revealed that WW domain binding protein 2 (WBP2) interacts with the WW domain, which enhances the cell transformation ability of YAP and TAZ [194,195,196,197]. Taken together, these results suggest that small molecules can be designed to target the WW domains of YAP and TAZ to inhibit their oncogenicity. Computational analyses predict that the cardiac glycoside digitoxin may bind to the first WW domain of YAP via the hydrophobic groove [145,146]. Interestingly, previous studies have shown that digitoxin exhibits cytotoxic effects against cancer cells [198], which makes it tempting to speculate that digitoxin’s anticancer effects could be due to abolishing the oncogenic activities of YAP and TAZ.

Although targeting the WW domains to inhibit YAP and TAZ appears promising, it is important to note that in some context, the WW domains inhibit the oncogenic properties of YAP and TAZ instead. For instance, in mammary epithelial cells, forced expression of YAP with mutations in the WW domains enhances cell migration and cell transformation when compared to cells expressing wildtype YAP [199]. Furthermore, several groups have reported that LATS1 and AMOTL1 interact with the WW domains of YAP, which could explain how the WW domains negatively regulate the activity of YAP [44,45,200]. Collectively, these observations suggest that the use of small molecule inhibitors to target the WW domains of YAP and TAZ for cancer therapy have to proceed with caution as these inhibitors may activate YAP and TAZ depending on the cell and tissue context.

### 7.5. Inhibiting Kinases

Earlier studies have reported that some kinases enhance YAP and TAZ activity. Since kinases are druggable by small molecules, the development of small molecules to inhibit kinases could be an attractive approach to suppress YAP and TAZ activity in cancer. YES proto-oncogene 1 (YES1), a non-receptor tyrosine kinase, is found to be crucial for the formation of a transcriptional complex that is required for β-catenin-driven cancers [21]. Proteins that are part of this transcriptional complex include YAP, β-catenin and TBX5. Importantly, the use of dasatinib, a small molecule inhibitor of YES1, inhibits the proliferation of cancer cell lines dependent on β-catenin activity [21]. Therefore, suppressing YES1 activity could be effective in cancers driven by aberrant YAP and β-catenin activity.

The addition of EGF to serum-starved mammary epithelial cells has been shown to inhibit YAP phosphorylation at serine 127, as well as promoting the nuclear localization of YAP [54]. These observations suggest that the EGFR signaling pathway could cross-talk with the Hippo signaling pathway. By screening a panel of small molecule inhibitors, Fan et al. (2013) have demonstrated that EGFR inhibits the Hippo signaling pathway and promotes the nuclear localization of YAP by activating PI3K and phosphoinositide-dependent kinase 1 (PDK1). In addition, PDK1 is observed to interact with the core Hippo pathway complex and that this complex dissociates when EGFR signaling is activated [54]. Crucially, treatment with PI3K and PDK1 inhibitors can inhibit the accumulation of YAP in the nucleus by EGF. Taken together, these results suggest that PI3K and PDK1 inhibitors could be used to inhibit YAP activity in cancer cells with active EGFR signaling. However, it is important to note that targeting the EGFR signaling pathway does not exclusively target Hippo signaling, because targeting EGFR signaling also affects other oncogenic and non-oncogenic cellular functions [201].

Targeting the core kinases of the Hippo signaling pathway, MST1/2 and LATS1/2, to activate YAP and TAZ activity could be desirable in regenerative medicine and in certain hematologic malignancies where YAP functions as a tumor suppressor. 9E1, a selective small molecule inhibitor of MST1, has been developed recently. 9E1 inhibits MST1 in the nanomolar range, and it can also inhibit endogenous MST1 kinase activity in cells [147]. It will be interesting to test whether 9E1 can be used as a therapeutic agent in hematologic malignancies and in regenerative medicine by upregulating YAP.

### 7.6. Modulating GPCR Signaling

As mentioned earlier, several extracellular ligands have been identified that can activate YAP and TAZ via GPCRs coupled to Gα_12/13_ or Gα_q/11_. Examples of these extracellular ligands include LPA, S1P and thrombin [47,48,49]. These observations suggest that the development of therapeutics to modulate GPCR signaling could be used to regulate the activity of YAP and TAZ for anticancer therapy and regenerative medicine. Similar to targeting EGFR signaling, targeting GPCR signaling also does not target Hippo signaling exclusively, because targeting GPCR signaling also affects multiple signaling pathways, as well.

Analogues of LPA that are less vulnerable to degradation by phosphatases and phospholipases have been developed to inhibit LPA receptors [202]. Another approach to block LPA signaling is to inhibit the biosynthesis of LPA. Autotaxin is an enzyme that is required for the biosynthesis of LPA [203]. A recent study has shown that palmitoyl α-bromomethylenephosphonate-1 (BrP-LPA) inhibits the synthesis of LPA by blocking the enzymatic activity of autotaxin. More importantly, treatment with BrP-LPA has been shown to suppress tumor growth in a lung cancer xenograft model [148].

To inhibit signaling mediated by S1P, a monoclonal antibody targeting S1P has been developed. Preclinical studies have shown that the use of this antibody can inhibit tumor growth, tumor invasion, as well as angiogenesis [204]. Blocking the production of S1P is another avenue to inhibit S1P signaling. Sphingosine kinase 1 (SPHK1) and sphingosine kinase 2 (SPHK2) are enzymes that catalyze the phosphorylation of sphingosine to produce S1P [205]. Therefore, the use of therapeutics to inhibit SPHK1 and SPHK2 could potentially reduce the level of S1P and inhibit the signaling pathway downstream of S1P receptors. ABC294640 is a small molecule inhibitor of SPHK2 that has been developed. It decreases S1P levels, suppresses the proliferation of cancer cells in vitro and inhibits tumor growth in immunocompromised mice [149].

### 7.7. Targeting the Mevalonate Pathway

The mevalonate pathway is an essential metabolic pathway that synthesizes isoprenoids, such as cholesterol, heme-A and dolichol [206]. Recent studies have demonstrated that the mevalonate pathway can enhance YAP and TAZ activity [150,151]. Mechanistically, the mevalonate pathway synthesizes geranylgeranyl pyrophosphate, which is essential for activating YAP and TAZ via Rho GTPases. More importantly, YAP and TAZ activities can be suppressed by using statins to inhibit the mevalonate pathway [150,151]. Taken together, these results demonstrated that statins could be used for cancer therapy to treat tumors with high YAP or TAZ activity.

### 7.8. miRNAs

miRNAs are a class of non-coding RNAs of 18–25 nucleotides in length that repress the expression of target genes by binding to the 3′-untranslated region of mRNAs. Previous studies have shown that miRNAs regulate numerous cellular processes, such as cell growth, cell death and cell differentiation [207,208,209]. Recent studies have demonstrated that some miRNAs play a role in tumorigenesis and disease progression in several cancer types by inactivating the Hippo signaling pathway.

Lin et al. (2013) have reported that miR-135b expression is elevated in highly-invasive lung cancer cell lines and that high levels of miR-135b can enhance lung cancer invasion and metastasis in xenograft mouse models [152]. Mechanistically, miR-135b promotes lung cancer metastasis by repressing genes within the Hippo signaling pathway, such as *LATS2*, *MOB1B* and *NDR2*. Crucially, the use of an antagomir against miR-135b is able to inhibit tumor growth and metastasis [152]. In another study, miR-130b is observed to be upregulated in glioblastoma. Functionally, upregulation of miR-130b can promote the maintenance of the cancer stem cell population [153]. mir-130b represses the Hippo pathway genes *MST1* and *SAV1*, resulting in the activation of YAP and TAZ. Importantly, the use of an antagomir against miR-130b inhibited the stem cell-like phenotype [153]. Taken together, results from these two studies suggest that miRNAs play a critical role to regulate the Hippo signaling pathway in cancer. Furthermore, oncogenic miRNAs that suppress Hippo pathway genes can potentially be targeted by antagomirs for cancer therapy.

MiRNAs also have therapeutic potential for cardiac regeneration, as well. Tian et al. (2015) showed that miR302-367 is essential for cardiomyocyte proliferation during development [154]. Using high-throughput sequencing of RNA derived from murine ESCs, the authors observed that miR302-367 target genes within the Hippo signaling pathway, such as *Mst1*, *Lats2* and *Mob1b*. These findings suggest that miR302-367 promotes cardiomyocyte proliferation by inhibiting the Hippo signaling pathway. Crucially, a brief treatment with miR-302 mimic can enhance cardiac regeneration in mice after injury [154]. These results imply that YAP and TAZ activity can be enhanced with miRNAs, although it remains to be determined whether the use of miRNAs will be effective in humans for regenerative medicine.

## 8. Conclusions

The downstream effectors of the Hippo signaling pathway, YAP and TAZ, are promising therapeutic targets for the treatment of cancer. To inhibit YAP and TAZ, most research is focused on the pharmacologic manipulation of signaling pathways that cross-talk with the Hippo pathway, as well as the development of compounds that disrupt the interactions between YAP and TEADs. However, there are other approaches to block YAP and TAZ activity. One approach that is not receiving as much attention is the development of therapeutics that inhibit the target genes of YAP and TAZ. This approach is promising because studies have shown that inhibiting some of these target genes can attenuate the oncogenic properties of YAP and TAZ [17,210]. Furthermore, some of the proteins regulated by YAP and TAZ are secreted (CTGF, CYR61, CXCL5), which make them more amenable for drug targeting by small molecules and neutralizing antibodies.

Although there is significant progress in the development of therapeutics to block the oncogenic effects of YAP and TAZ in cancer, more work is required to develop new drugs to target the Hippo signaling pathway for tissue regeneration. The development of selective inhibitors that target MST1/2 or LATS1/2, such as the drug 9E1, is one potential approach to activate YAP and TAZ in regenerative medicine. However, great care is required to activate YAP and TAZ transiently because prolonged activation of YAP and TAZ may lead to the development of malignant tumors. There is great promise for the development of miRNA-based therapeutics, such as the miR-302 mimic, for regenerative medicine. However, significant hurdles, such as delivery strategies and off-target effects, need to be overcome before miRNAs can be used for therapeutic purposes in humans.

## Figures and Tables

**Table 1:** Therapeutic approaches to regulate YAP/TAZ activity.

Compound(s)	Mechanism	References
Dasatinib	Inhibits YES1	[21]
Verteporfin, flufenamic acid	Disrupt YAP-TEAD interaction	[133,134]
YAP-like and VGLL4-like peptides	Disrupt YAP-TEAD interaction	[135,136,137]
XAV939, Inhibitor of WNT response 1 (IWR-1), G007-LK	Inhibit tankyrases to stabilize AMOTs	[138,139,140]
093G9	Monoclonal antibody against CYR61	[141]
FG-3019	Monoclonal antibody against CTGF	[142]
Foretinib, sunitinib	Small molecule inhibitors of AXL	[143]
Navitoclax (ABT-263)	Small molecule inhibitor of BCL-XL	[144]
Digitoxin	May inhibit WBP2-YAP interaction	[145,146]
9E1	Small molecule inhibitor of MST1	[147]
BrP-LPA	Inhibits synthesis of LPA	[148]
ABC294640	Small molecule inhibitor of SPHK2	[149]
Statins	Inhibit mevalonate pathway	[150,151]
Antagomir against miR-135b	Derepression of *LATS2*, *MOB1B*	[152]
Antagomir against miR-130b	Derepression of *MST1*, *SAV1*	[153]
MiR-302 mimic	Repression of *MST1*, *LATS2*, *MOB1B*	[154]
